# Are Foods from the COVID-19 Pandemic Lockdown Low in Nutrients? An Analysis of Chinese Psychological Distress Effects

**DOI:** 10.3390/nu14214702

**Published:** 2022-11-07

**Authors:** Wen Jiao, Yu-Tao Xiang, Angela Chang

**Affiliations:** 1 Department of Communication, Faculty of Social Sciences, University of Macau, Macao 999078, China; 2 Unit of Psychiatry, Institute of Translational Medicine, Faculty of Health Sciences, University of Macau, Macao 999078, China; 3 Center for Cognition and Brain Sciences, University of Macau, Macao 999078, China; 4 Institute of Communication and Health, Lugano University, 6900 Lugano, Switzerland

**Keywords:** COVID-19, lockdown, nutrient intake, psychological distress, food advice, mediated

## Abstract

Background: The city-wide COVID-19 lockdown has resulted in psychological anguish, which may have an impact on dietary consumption. This study’s dual goals are to show how Chinese food consumption was altered before and after the lockdown, and to examine the nutrient density for the psychologically affected group. Methods: A cross-sectional study involving 652 people from Mainland China, Taiwan, and Macao was conducted with the aid of a web-based questionnaire. Sociodemographic characteristics, related environmental factors, nutrient consumption, food recommendations, and psychological distress were all measured. 516 trustworthy data revealed that two nutrient-poor foods were consumed less frequently during the lockdown than they were before to the COVID-19 outbreak (i.e., salty snacks and alcoholic beverages). People who endured high levels of psychological distress in particular tended to consume more. Particularly, those who experienced high levels of psychological distress had a tendency to consume far more alcohol than people who only experienced low levels of stress. Comparing the time before the COVID-19 to the present, there has statistically been an increase in the frequency of family members recommending diets. According to research, by food advice, individuals who experience psychological distress should consume more nutrient-dense foods (78.7%) than nutrient-poor ones (61.9%). Thus, food advice plays a role in mediating the relationship between psychological distress and dietary decisions for nutrient-rich (*b* = 0.186, *p* < 0.001) or nutrient-poor (*b* = 0.187, *p* < 0.001) food groups. This study provides insights for lowering psychological distress through dietary consumption, where the exact mechanisms underlying these connections have not been thoroughly elucidated. It encourages nutrition research by recommending practical nutrition education from family and environmental activities. Chronic psychological anguish may have a crucial relationship to secure access to food and a balanced diet. Along with nutrition instruction, it is critical to develop skills in interventions such as food procurement and culinary knowledge.

## 1. Introduction

An unparalleled threat to world public health has emerged with the COVID-19 pandemic epidemic. Regional or national lockdowns are imminent, due to the virus’s rapid spread, to ensure there is little to no human contact, to lower the total infection rate, and to avoid overtaxing the social health system. However, food supply systems during a lockdown might suffer from a breakdown, leaving the population with fewer nutritious food options, and a change in dietary preferences [1,2]. Concomitantly, closure measures, including shutting down workplaces and schools, outlawing non-essential restaurant dine-in, and forbidding private gatherings, may have altered how individuals obtained food, as well as where and how they prepared it [3,4,5,6].

Existing research suggested that during a lockdown, people tended to eat more nutrient-poor meals such snacks, desserts, alcoholic beverages, and sugary beverages [7,8,9,10,11,12]. The increased intake of products with low nutritional value, such as ultra-processed foods, was in turn associated with psychological distress in Italy [13] and Chile [14]. Since these nutritionally deficient meals have been related to a number of various health concerns, such as a high risk of obesity, diabetes, dementia, and chronic disease [15,16,17], it was not their low nutritional value that worried us, given that comfort foods, which are heavy in carbs and are used to relieve chronic psychological distress, are associated with unhealthy eating and food insecurity.

Contrarily, nutrient-dense food consumption is strongly advised, because the value of recommended nutrient intake is large and is thought to satisfy the needs of almost all healthy individuals. A variety of fruits and vegetables with fiber, vitamins, and minerals are included in the daily nutrient intake, as well as whole grains with fewer processed carbohydrates, low-fat or fat-free dairy products, eggs, shellfish, legume products, unsalted nuts, and white meat for protein [18,19,20,21,22,23]. In conclusion, a balanced diet that provides enough nourishment is good for people’s health and wellbeing. Therefore, it is crucial to investigate the function of nutritional psychiatry in reducing the worry, tension, and sadness that the COVID-19 pandemic has caused.

Making dietary decisions to maximize the chance of nutritional food consumption frequently involves seeking out food advice [24,25]. According to two works of research on Chinese immigrants living abroad, family members’ opinions on food were among the most important elements in determining a healthy diet [26,27]. Consumers in Beijing were found to be more interested in nutrition information and had a tendency to eat healthier, according to a cross-sectional survey [24]. Given that information-seeking behavior is related to food consumption, while psychological distress is linked to the consumption of energy-dense and low-nutrient foods, research questions (RQ) were proposed to learn how the lockdown induced psychological distress, as well as discern its impact on nutrient-dense and low-nutrient diets. They included the following: 

RQ1: Did the lockdown have any effects on people who reported dietary changes before and during it?

RQ2: How did people’s eating habits and methods for getting nutritional advice change as a result of the COVID-19 pandemic lockdown? 

RQ3: How did psychological distress affect food intake during the COVID-19 pandemic lockdown?

RQ4: How did food advice during the COVID-19 pandemic lockdown influence the link between psychological distress and the intake of nutrient-dense and nutrient-poor foods?

## 2. Materials and Methods

The Corona Cooking Survey, an international project, served as the basis for developing the questionnaire. It sought to compare how the COVID-19 pandemic lockdown affected food intake and type, as well as compare media usage across 38 nations [3,4,5]. The questionnaire was jointly created, and all research participants endorsed the final version. The Institutional Review Board at the University of Antwerp (UAntwerp) in Belgium centrally approved the study’s ethical conduct, because the initiative was begun by UAntwerp academics (Approval Code: SHW_20_46).

Because the virus outbreak was first reported in China [25,28] and because symptoms of psychological distress increased throughout the COVID-19 pandemic outbreak [29,30], this study concentrated on the Chinese context. From April to June of 2020, an online poll was conducted to examine the effects of psychological strain on Chinese health. We gathered adult respondents from Macao, Taiwan, and Mainland China.

### 2.1. Procedure

Two versions of the English questionnaire were translated into Chinese (simplified and traditional Chinese), and the back translation was carried out by native speakers. The length of the COVID-19 pandemic lockdown, as well as shopping, cooking, food advice seeking, and dietary decisions before and after, were all measured. The questionnaire’s items could not be changed or added for the sake of consistency. All data collection procedures used the university-supported Qualtrics XM platform (Qualtrics, Provo, UT, USA).

A primary data source nominated additional possible data sources to participate in the research investigations using the snowball sampling technique. A pilot test was conducted by the researchers to evaluate the usefulness, time requirements, and potential for improvement of the questionnaire. To get as many respondents as possible, the Chinese poll was promoted and made available on regional social media sites like WeChat, QQ, Lines, and Sina Weibo. 652 Chinese people expressed interest in taking part in this online poll.

By signing the consent form at the start of the survey, respondents were educated about the survey’s goals and rights. Participation was anonymous and voluntary. The survey questionnaire took, on average, at least 35 min to complete. Adults who were at least 18 years old, spoke Chinese, and who had lived in either Mainland China, Taiwan, or Macao since the COVID-19 outbreak began were required to meet the inclusion criteria. To boost the return rate, a reward was provided to potential responders who completed the survey. Twenty participants each received a $15 USD prize through a random selection.

### 2.2. Measurements

Nineteen questions about food type and frequency of consumption related to nutrient intake were included before and during the COVID-19 pandemic lockdown [4,9,22,31,32]. A 7-point Likert scale was used to score each question (1 = hardly ever; 7 = twice or more a day). Fruits, vegetables, beans/legumes, unsalted nuts, unprocessed fish, chicken, red meat, vegetarian substitutes, whole grains, milk, other dairy products, plant-based beverages, and non-sugar-based beverages were among the thirteen categories of nutrient-dense foods. A greater percentage of nutrient-dense food intake was indicated by a higher average score. In contrast, six nutrient-poor foods were white wheat, sugary drinks, alcoholic beverages, sweet/salty snacks, and processed meat. A higher average result meant that the amount of nutrient-poor food consumed was higher.

Six standard questions were used to gauge reported symptoms of psychological distress during times of stress [33,34], including Hopelessness, restlessness, everything that needs to be done, a sense of worthlessness, tension, and sadness. A 7-point Likert scale was used to rate each item (1 = never; 7 = often) (Cronbach’s alpha = 0.891). A higher average score indicated greater psychological suffering during the first COVID-19 lockdown.

Eight standard questions about information sources for food guidance and regularity of getting trustworthy recommendations for a healthy diet were used to gauge the effectiveness of food advice [24,27,35]. Each item was scored on a 7-point Likert scale (1 = never; 7 = always needed/wanted). Family members, friends, experts (such as dietitians, nutritionists, medical physicians, and scientists), and celebrities (such as celebrity chefs, celebrities, and food influencers) were the four main groups (Cronbach’s alpha = 0.932). A higher average score suggested a higher level of heeding dietary instructions.

Environmental factors associated with COVID-19 and sociodemographic traits were used as controls. Gender, age, location, education, and employment status were taken into account for sociodemographic characteristics, whereas COVID-19-related variables included income loss as a result of COVID-19, financial hardships when buying food, severity of closure measures, and self-reported lockdown time [3,4,5]. For illustration, one question looked at how frequently a person struggled to have enough money to buy food (1 = never; 7 = every time I went grocery shopping). Additionally, fifteen lockdown-related multiple-choice questions were included (where 0 represented a no answer and 1 a yes answer). An increased average score denoted a greater degree of closure. The Supplementary Materials File S1 contain a list of all pertinent measurements and scales.

### 2.3. Data Processing

Data processing was done using SPSS Statistics 24 (IBM Corporation, Armonk, NY, USA). To look into the demographics of the samples, a descriptive analysis was done. The two patterns of nutrient consumption and the four sources of food advice were compared before and during the COVID-19 pandemic lockdown using a paired-sample *t*-test with a 95% confidence interval (CI). The middle 50% of observations in a box-and-whisker diagram were used to compare distributions of nutrient-poor food intake in Mainland China, Macao, and Taiwan.

The chi-square test of independence was used to evaluate the relationship between psychological distress in high and low groups and food intake in high and low groups during lockdowns after recoding the 7-point scale into low (1 to 4) and high (5 to 7) groups. The odds ratio (OR) was also calculated to demonstrate the degree to which the test group’s (high psychological distress) consumption of the targeted foods exceeded that of the control group’s (low psychological distress) [36].

Additionally, multiple linear regressions examined the mediating role of dietary recommendations on the associations between psychological distress (an independent variable) and nutrient-dense and nutrient-poor food intake during the pandemic lockdown (dependent variables). The regression model received all variables as continuous inputs. The ratio of the mediation effect through dietary advice to the total effect was used to calculate the relative magnitude of the effects [37]. The statistical level was set at 0.05 (two-tailed).

## 3. Results

### 3.1. Descriptive Analysis

516 valid and finished responses were assessed as a result. A total of 85.5% of the respondents (*n* = 441) were from Mainland China, with the remaining 14.5% (*n* = 75) coming from Taiwan and Macao. There were more female respondents than male respondents (62.4%, *n* = 322), and the respondents’ ages ranged from 18 to 79 (M = 30.99, SD = 11.33). A bachelor’s degree was the most common degree possessed by respondents (41.5%, *n* = 214), followed by a high school diploma or its equivalent (30.0%, *n* = 155). The lockdown time lasted 10.1 weeks on average (SD = 9.28). The reported financial hardship for food purchases was moderate (M = 2.66, SD = 1.44), while the unemployment rate rose to 30.0% (*n* = 155), and income loss was 53.5% (*n* = 276).

### 3.2. Food Intake before and during the COVID-19 Lockdown

Using paired-sample *t*-tests, nineteen types of food intake were identified among Chinese respondents before and during the lockdown. Under the category of nutrient-poor foods, the decreased intake of salty snacks during the lockdown (M = 3.98, SD = 1.57) was found, by comparing it to consumption before the lockdown (M = 4.10, SD = 1.56), with statistically significant results (*t_515_* = −2.459, *p* = 0.014, 95% CI [−0.220, −0.025]). Similarly, the consumption of alcoholic beverages was lower during the lockdown (M = 3.59, SD = 1.86) than before it (M = 3.68, SD = 1.86), and showed statistical significance (*t_515_* = −2.002, *p* = 0.046, 95% CI [−0.184, −0.002]). In comparison, the remaining seventeen food categories did not differ significantly in intake frequency during the lockdown. The reported consumption of food nutrition during the lockdown was more fresh vegetables (M = 5.02, SD = 1.36), fruits (M = 4.96, SD = 1.53), non-sugared beverages (M = 5.00, SD = 1.64), milk (M = 4.49, SD = 1.52), and legumes (M = 4.43, SD = 1.41). The comparison of mean differences in food intake before and during the COVID-19 pandemic is tabulated in Table A1 in Appendix A.

A box-and-whisker diagram was created to identify the range of values with the middle 50% of nutrient-poor food intake during the COVID-19 pandemic lockdown in order to avoid the potential effects of extreme values. The same variety of processed meat, sweets, white wheat, and sugared beverages was consumed by 50% of the respondents in Mainland China (Median = 4.00, Lower Quartile (Q1) = 3, Upper Quartile (Q3) = 6, IQR = 3). They ingested salty snacks in a more concentrated way with a lower range (Median = 4.00, Q1 = 3, Q3 = 5, IQR = 2).

**Figure 1:**
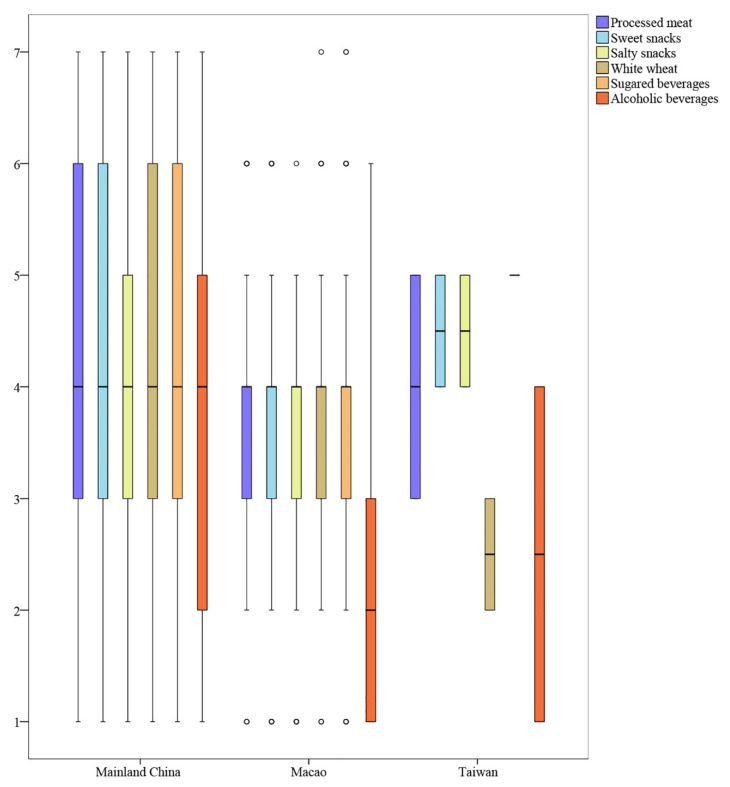
Clustered boxplot depicting the concentrated distribution of nutrient-poor food intake in Mainland China, Macao, and Taiwan.

In comparison to the other categories of nutrient-poor foods, the distribution of alcohol intake in Macao was more discontinuous (Median = 2.00, Q1 = 1, Q3 = 3, IQR = 2) than it was for the other groups of foods (Median = 4.00, Q1 = 3, Q3 = 4, IQR = 1). Taiwan respondents preferred to consume less white wheat (Median = 2.50, Q1 = 2, Q3 = 3, IQR = 1) and more salty and sweet snacks (Median = 4.50, Q1 = 4, Q3 = 5, IQR = 1). There was no discernible variation in the respondents in Taiwan’s consumption of sweetened beverages. The concentrated distribution of nutrient-poor food intake in Mainland China, Macao, and Taiwan is shown in a clustered boxplot in Figure 1.

### 3.3. Food Advice before and during the COVID-19 Lockdown

There was a statistically significant difference in the amount of people seeking dietary advice during the lockdown (M = 4.33, SD = 1.25) and before it (M = 4.29, SD = 1.22) (*t_515_* = 2.277, *p* = 0.023, 95% CI [0.006, 0.082]). To be more precise, family members’ recommendations for meals during the lockdown were better (M = 4.82, SD = 1.47) than they had been previously (M = 4.75, SD = 1.50), and showed statistical significance (*t_515_* = 1.978, *p* = 0.049, 95% CI [0.000, 0.135]). There was no difference in the information from friends, experts, or famous people. The paired *t*-test was employed in Table 1 to compare food advice from various sources before and during the COVID-19 pandemic lockdown.

### 3.4. Psychological Distress and Nutrient Intake during the COVID-19 Lockdown

A total of 204 respondents (39.5%) reported experiencing severe psychological distress during the COVID-19 lockdown (M = 3.63, SD = 1.43), while 312 respondents (60.5%) had minimal psychological hardship. A chi-square test for independence was conducted to investigate the link between psychological distress (high, low) and nutrient-dense foods (high, low), and a *p*-value of 0.05 or below indicated statistical significance. One of the findings showed that individuals with varying degrees of psychological distress were more inclined than their counterparts to consume more nutrient-dense foods while under lockdown.

According to cross-tabulation analysis, people in the high psychological distress group consumed low-fat sources of protein (unprocessed fish) at a rate that was 4.4 times higher than people in the low psychological distress group (χ^2^ = 62.872, *p* < 0.001). The consumption of legumes (χ^2^ = 49.314, *p* < 0.001) and food categories high in low-fat protein (unprocessed vegetarian alternatives) also revealed a similar tendency (χ^2^ = 56.932, *p* < 0.001). The examination of nutrient-dense food intake during the COVID-19 pandemic lockdown between high and low psychological distress is shown in Table 2.

The intake of nutrient-poor foods was reported by participants who were experiencing psychological distress. This included processed meat (χ^2^ = 57.660, *p* < 0.001), sweet snacks (χ^2^ = 52.549, *p* < 0.001), salty snacks (χ^2^ = 67.230, *p* < 0.001), white wheat (χ^2^ = 72.186, *p* < 0.001), and sugared beverages (χ^2^ = 58.987, *p* < 0.001). Individuals with significant psychological anguish tended to consume more alcoholic beverages (five times more) than those with moderate psychological distress (OR = 5.110). There was a strong correlation between psychological distress and alcohol use (χ^2^ = 72.056, *p* < 0.001). Likewise, compared to their peers, those who reported significant levels of psychological distress ate white wheat with large amounts of processed carbs (OR = 4.966) and salty snacks (OR = 4.715). Table 3 shows the crosstab analysis of the consumption of nutrient-poor foods with either high or low psychological distress conditions during the COVID-19 pandemic.

### 3.5. Link between Psychological Distress and Nutrient-Dense or Nutrient-Poor Foods

To determine the effect of psychological distress on nutritional consumption patterns through dietary advice, a multiple regression analysis was carried out. A non-standard effect (*b*) demonstrated how the independent variable affected the variables. Psychological distress had a statistically significant impact on food recommendations (*b* = 0.298, *p* < 0.001), which in turn influenced nutrient-dense food intake (*b* = 0.623, *p* < 0.001).

According to the regression models, certain sociodemographic factors, including gender, geography, age, and employment status, could accurately predict how much food advice and nutrient-poor food people would consume during the lockdown. In particular, respondents who were employed (*b* = 0.233, *p* = 0.033), lived in Mainland China (*b* = 0.793, *p* < 0.001), and were younger (*b* = −0.830, *p* = 0.044) were more likely than their peers to listen to dietary advice. Additionally, men and those with jobs ate more nutrient-poor meals than their counterparts (*b* = 0.190, *p* = 0.025 and *b* = 0.229, *p* = 0.011, respectively). Regarding the COVID-19-related characteristics, closure measures also had a negative impact on the consumption of nutrient-poor foods (*b* = −0.354, *p* = 0.034), showing that respondents who experienced less closure measures were more likely to consume foods that were low in nutrients. The multiple regression analysis between psychological distress, food advice, and nutrient intake patterns is shown in Table 4.

In line with Table 4, a model showed the connection between psychological distress and nutrient-dense food intake that was mediated by food recommendations (*b* = 0.186, *p* < 0.001). Food advice significantly mediated the relationship between psychological distress and nutrient-poor food intake (*b* = 0.187, *p* < 0.001). Additionally, psychological distress significantly affected food advice (*b* = 0.298, *p* < 0.001), and food advice predicted the nutrient-poor food intake (*b* = 0.627, *p* < 0.001).

**Figure 2:**
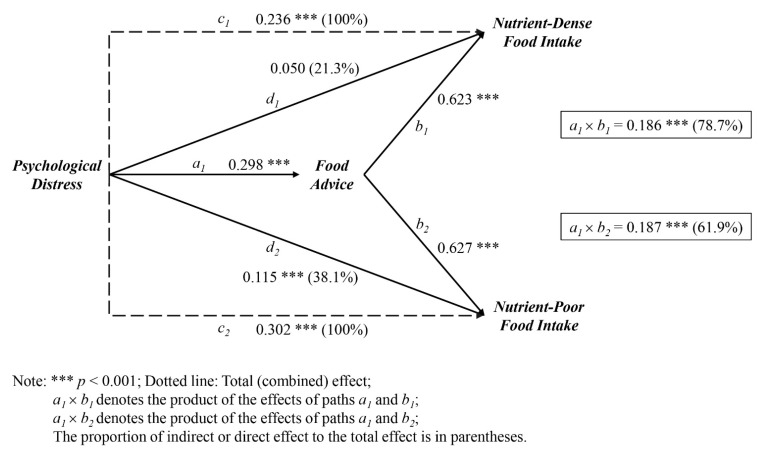
Psychological distress during the COVID-19 pandemic lockdown and its effect on nutrient intake patterns through food recommendations.

Overall, psychological distress had a substantial positive effect on nutrient-dense food intake (*b* = 0.236, *p* < 0.001). Meal recommendations mediated 78.7% of the overall impact of psychological distress on nutrient-dense food intake. Similar to the effect of psychological distress on nutrient-dense food intake, nutrient-poor food intake was positively predicted (*b* = 0.302, *p* < 0.001). The total impact of psychological distress on the use of nutrient-poor foods was 61.9% indirect, or through food advice, and 38.1% direct, or through other means. Figure 2 shows how psychological distress during the COVID-19 pandemic lockdown affected nutrient intake patterns through food recommendations.

## 4. Discussion

For the body to restore its nutrients, a balanced diet is crucial. During the COVID-19 pandemic lockdown, Chinese consumption of salty snacks and alcoholic beverages declined statistically by 2.9% and 2.4%, respectively. This study confirmed the idea that during the pandemic lockdown, Chinese people emphasized nutrition and food-based dietary guidelines, which was consistent with other findings (e.g., [22,24]). Individuals can develop and maintain healthy eating habits, stay physically fit, and even lower their risk of underlying diseases, such as obesity and cardiovascular disease, by consuming adequate amounts of dietary fiber, vitamins, and minerals (i.e., fruits and vegetables) and limiting their intake of sugary beverages (by replacing them with sugar-free beverages) [7,9,16,19,38].

### 4.1. Lockdown Impacts on Dietary Changes for Chinese in Mainland China, Macao, and Taiwan

During the lockdown, alcohol consumption was more pronounced in Macao than in Taiwan, where sweet and salty delicacies were more popular. Comparatively speaking, people in Mainland China had more access to nutrient-poor diets than those in Macao and Taiwan. During the COVID-19 epidemic in 2020, there were no tight city-wide lockdowns in Taiwan or Macao. As a result, the lockdown level may have played a significant role in accurately portraying nutrient intake.

Chinese respondents who sought advice on what to eat during the COVID-19 lockdown most frequently did so from family members, with a 1.5% increase in frequency over the previous months. Specifically, people from Mainland China sought family advice on food more frequently than people from other geographic locations. The results conflicted with other research that indicated that the Internet was a source of information during the lockdown (e.g., [25,35,39,40,41]. It is interesting to notice that, for Chinese people, family members were more influential than friends, acquaintances, experts, or public figures.

### 4.2. Nutritional Advice and Education

Previous studies have shown that seeking nutritional advice has been crucial in encouraging nutrient-dense food intake (e.g., [24,35,41]). It encourages nutrition studies by suggesting realistic nutrition communication from family and environmental activities. Chronic psychological distress may be a crucial relationship between secure access to food and healthy eating. Along with nutrition instruction, it is crucial to develop skills in interventions, such as food procurement and culinary knowledge.

Chinese people who listen to dietary recommendations may be able to better connect psychological distress with nutrient-dense food consumption while avoiding nutrient-poor food intake. People with severe psychological distress might learn more about nutrition and food. The evidence was in favor of the idea that people with psychological disturbances intuitively sought the help of loved ones in an effort to reduce their symptoms and regain their diet-related health. As a result, receiving food recommendations from close friends and family members may strengthen a person’s resolve and inspire them to take action that will improve their health, by giving the impression that they have access to social support [26,35].

### 4.3. Implications and Limitations

This study had multiple ramifications, the first of which was that people who were experiencing psychological distress could recover if they maintained a balanced diet and nutritional intake. The value of human connections must be emphasized, notably the value of asking family members for dietary recommendations. Education on the planning, management, selection, preparation, cooking, and consumption of nutritious food by competent authorities and managers is crucial for people, whether they seek or give food advice to improve food literacy. Secondly, the connection between psychological distress and dietary choices for food groups of nutrient-dense or nutrient-poor was mediated in part by food advice. Since the processes behind these relationships have not been precisely established, this study offers insights for reducing psychological distress through dietary intake.

There are a few restrictions to be aware of. For instance, unconscientious responses could have been produced by the standardized questionnaire. Despite the researcher’s best efforts to elicit thoughtful responses, there was no way to verify whether the respondent actually understood the question or carefully read it before responding. Particularly, because surveys are thought to be too lengthy, respondent tiredness most likely occurred during the survey. Unfortunately, a low completion rate was discovered to be a sign of survey-taking tiredness. Although the disadvantage is difficult to overcome, it is suggested that future surveys concentrate on asking questions that are brief and basic, because they are more likely to yield the most accurate answers.

The convenient sample, which was not chosen by random selection, was another drawback. As a result, it is impossible for the study’s sample to accurately reflect the Chinese population under investigation. This limits our capacity to extrapolate findings from our sample to the relevant population. Therefore, to see if there are variances from this study, future research should use random sampling.

## 5. Conclusions

This study provided insight into how to use dietary recommendations during the COVID-19 pandemic lockdown to help the general population who were experiencing psychological distress transitions from nutrient-poor to nutrient-dense food intake. In particular, compared to before the global health crisis, the Chinese population consumed much less alcohol and salty food. The frequency of mental anguish increased. The likelihood of alcohol abuse was highest in those who experienced significant levels of psychological distress. Family members were also the most frequently requested source of information on food. The association between psychological distress and nutrient-dense versus nutrient-poor food intake was found to be much more mediated by food recommendations, which supported a positive impact on people’s psychological health. This study highlighted the important contribution of information produced from close family relationships, and offered new suggestions for preventing mental health issues through nutrient-dense foods in the midst of serious health emergencies. This study also provided a fresh perspective on the model mechanisms relating psychological factors to behavioral results.

## Figures and Tables

**Table 1:** Comparison of mean differences between sources of food advice before and during the COVID-19 pandemic lockdown using the paired *t*-test.

Source	M (SD)			95% CI		*t*_515_
	During	Before	Difference	Lower	Upper	
Food advice	4.33 (1.25)	4.29 (1.22)	0.04 (0.44)	0.006	0.082	2.277 *
Family members	4.82 (1.47)	4.75 (1.50)	0.07 (0.78)	0.000	0.135	1.978 *
Friends	4.34 (1.45)	4.32 (1.37)	0.02 (0.93)	−0.057	0.104	0.567
Experts	4.16 (1.54)	4.13 (1.50)	0.03 (0.63)	−0.025	0.084	1.071
Celebrities	4.01 (1.56)	3.95 (1.53)	0.05 (0.69)	−0.005	0.115	1.799

Note: * *p* < 0.05.

**Table 2:** Crosstab analysis of nutrient-dense food intake between high and low psychological distress during the first COVID-19 pandemic lockdown.

Nutrient-Dense Foods	Psychological Distress		χ^2^	OR
	Low Level	High Level		
	312 (%)	204 (%)		
Fruit				
Low intake	140 (44.9)	63 (30.9)	10.116 **	1.822
High intake	172 (55.1)	141 (69.1)		
Vegetables				
Low intake	125 (40.1)	66 (32.4)	3.146	-
High intake	187 (59.9)	138 (67.6)		
Legumes/pulses				
Low intake	204 (65.4)	69 (33.8)	49.314 ***	3.696
High intake	108 (34.6)	135 (66.2)		
Unsalted nuts or nut spread				
Low intake	204 (65.4)	93 (45.6)	19.788 ***	2.254
High intake	108 (34.6)	111 (54.4)		
Unprocessed fish				
Low intake	230 (73.7)	79 (38.7)	62.872 ***	4.438
High intake	82 (26.3)	125 (61.3)		
Unprocessed poultry				
Low intake	217 (69.6)	82 (40.2)	43.619 ***	3.398
High intake	95 (30.4)	122 (59.8)		
Unprocessed red meat				
Low intake	201 (64.4)	91 (44.6)	19.715 ***	2.249
High intake	111 (35.6)	113 (55.4)		
Unprocessed vegetarian alternative				
Low intake	221 (70.8)	76 (37.3)	56.932 ***	4.090
High intake	91 (29.2)	128 (62.7)		
Whole wheat				
Low intake	215 (68.9)	90 (44.1)	31.369 ***	2.808
High intake	97 (31.1)	114 (55.9)		
Milk				
Low intake	191 (61.2)	77 (37.7)	27.226 ***	2.604
High intake	121 (38.8)	127 (62.3)		
Other dairy products				
Low intake	202 (64.7)	71 (34.8)	44.377 ***	3.440
High intake	110 (35.3)	133 (65.2)		
Plant-based drinks				
Low intake	223 (71.5)	96 (47.1)	31.154 ***	2.819
High intake	89 (28.5)	108 (52.9)		
Non-sugared beverages				
Low intake	141 (45.2)	58 (28.4)	14.626 ***	2.076
High intake	171 (54.8)	146 (71.6)		

Note: ** *p* < 0.01, *** *p* < 0.001.

**Table 3:** Crosstab analysis of nutrient-poor food intake between high and low psychological distress during the COVID-19 pandemic lockdown.

Nutrient-Poor Foods	Psychological Distress		χ^2^	OR
	Low Level	High Level		
	312 (%)	204 (%)		
Processed meat				
Low intake	223 (71.5)	77 (37.7)	57.660 ***	4.133
High intake	89 (28.5)	127 (62.3)		
Sweet snacks				
Low intake	224 (71.8)	81 (39.7)	52.549 ***	3.865
High intake	88 (28.2)	123 (60.3)		
Salty snacks				
Low intake	236 (75.6)	81 (39.7)	67.230 ***	4.715
High intake	76 (24.4)	123 (60.3)		
White wheat				
Low intake	221 (70.8)	67 (32.8)	72.186 ***	4.966
High intake	91 (29.2)	137 (67.2)		
Sugared beverages				
Low intake	220 (70.5)	74 (36.3)	58.987 ***	4.201
High intake	92 (29.5)	130 (63.7)		
Alcoholic beverages				
Low intake	247 (79.2)	87 (42.6)	72.056 ***	5.110
High intake	65 (20.8)	117 (57.4)		

Note: *** *p* < 0.001.

**Table 4:** Multiple regression analysis between psychological distress, food advice, and nutrient intake patterns during the COVID-19 pandemic lockdown.

	Unstandardized Effect (*b*)				
Variable	Food Advice	Nutrient-Dense Foods	Nutrient-Dense Foods ^T^	Nutrient-Poor Foods	Nutrient-Poor Foods ^T^
Control block					
Gender	−0.085	0.073	0.020	0.190 *	0.137
Age ^a^	−0.830 *	0.250	−0.267	−0.562	−1.083 *
Geographic location	0.793 ***	0.018	0.512 ***	0.043	0.540 ***
Education	−0.082	0.040	−0.011	−0.026	−0.077
Employment status	0.233 *	−0.012	0.134	0.229 *	0.375 ***
Income loss	0.053	0.065	0.098	−0.028	0.005
Financial difficulties with food	0.018	0.009	0.020	0.045	0.056
Degree of closure measures	0.186	−0.073	0.043	−0.354 *	−0.237
Lockdown time ^a^	−0.240	−0.008	−0.158	−0.154	−0.305 *
Prediction block					
Psychological distress	0.298 ***	0.050	0.236 ***	0.115 ***	0.302 ***
Food advice	-	0.623 ***	-	0.627 ***	-
Explanatory power					
*R*-squared	0.248	0.563	0.168	0.556	0.296
*F*-value	16.675 ***	59.090 ***	10.189 ***	57.455 ***	21.247 ***

Note: * *p* < 0.05, *** *p* < 0.001; ^a^ denotes transformation by *lg* when entering regressions; ^T^ for total effect regression model.

## Data Availability

Derived data supporting the findings of this study are available from the corresponding author (Angela Chang) upon reasonable request.

## Supplementary Materials

The following supporting information can be downloaded at: https://www.mdpi.com/article/10.3390/nu14214702/s1, File S1: Detailed measurements and scales of variables.

## Appendix A

**Table A1:** Comparison of mean differences in food intake before and during the COVID-19 pandemic using the paired *t*-test.

	M (SD)			95% CI		*t*_515_
Category	During	Before	Difference	Lower	Upper	
Nutrient-dense foods						
Fruit	4.96 (1.53)	4.94 (1.48)	0.02 (0.90)	−0.060	0.095	0.440
Vegetables	5.02 (1.36)	5.07 (1.38)	−0.05 (0.93)	−0.133	0.028	−1.272
Legumes/pulses	4.43 (1.41)	4.47 (1.34)	−0.04 (1.04)	−0.130	0.049	−0.891
Unsalted nuts or nut spread	4.19 (1.68)	4.21 (1.57)	−0.03 (1.09)	−0.120	0.069	−0.525
Unprocessed fish	4.14 (1.51)	4.12 (1.54)	0.03 (0.94)	−0.056	0.106	0.609
Unprocessed poultry	4.20 (1.54)	4.15 (1.55)	0.05 (1.02)	−0.036	0.141	1.166
Unprocessed red meat	4.28 (1.56)	4.34 (1.57)	−0.07 (1.07)	−0.162	0.023	−1.481
Unprocessed vegetarian alternative	4.30 (1.61)	4.27 (1.60)	0.03 (1.18)	−0.067	0.137	0.670
Whole wheat	4.19 (1.58)	4.29 (1.52)	−0.10 (1.21)	−0.203	0.006	−1.861
Milk	4.49 (1.52)	4.45 (1.47)	0.04 (1.01)	−0.051	0.125	0.824
Other dairy products	4.37 (1.56)	4.36 (1.56)	0.02 (1.14)	−0.083	0.114	0.309
Plant-based drinks	4.10 (1.64)	4.13 (1.66)	−0.03 (1.16)	−0.131	0.069	−0.609
Non-sugared beverages	5.00 (1.64)	4.97 (1.63)	0.03 (1.08)	−0.062	0.124	0.652
Nutrient-poor foods						
Processed meat	4.26 (1.62)	4.29 (1.64)	−0.04 (1.11)	−0.133	0.059	−0.752
Sweet snacks	4.16 (1.57)	4.24 (1.48)	−0.08 (1.19)	−0.182	0.023	−1.520
Salty snacks	3.98 (1.57)	4.10 (1.56)	−0.12 (1.13)	−0.220	−0.025	−2.459 *
White wheat	4.28 (1.66)	4.28 (1.60)	0.01 (1.18)	−0.096	0.108	0.112
Sugared beverages	4.18 (1.63)	4.13 (1.56)	0.05 (1.08)	−0.043	0.144	1.058
Alcoholic beverages	3.59 (1.86)	3.68 (1.86)	−0.09 (1.06)	−0.184	−0.002	−2.002 *

Note: * *p* < 0.05.
