# Supplementary material for: Are Foods from the COVID-19 Pandemic Lockdown Low in Nutrients? An Analysis of Chinese Psychological Distress Effects
Source: Nutrients. 2022 Nov 7;14(21):4702. doi: 10.3390/nu14214702 (PMC9656422; doi:10.3390/nu14214702)
Supplement: Supplementary file 1 [file nutrients-14-04702-s001.zip › Supplementary File S1.pdf]

## Supplementary Materials

## Measurements

---

### Psychological Distress
Question: How do you feel during the COVID-19 pandemic? Since the lockdown,
(Likert scale: 1 = Never; 7 = All the time)
- I feel hopeless
- I feel restless or fidgety
- I feel that everything requires effort
- I feel worthless
- I feel nervous
- I feel so depressed that nothing could cheer me up

### Nutrient Intakes before or during the COVID-19 Lockdown
Question: How often did you eat the following (portions of) foods?
(1 = Almost never; 7 = 2x or more times a day)

*Nutrient-Dense Food Intake*
- Fruit (fresh or frozen)
- Vegetables (fresh or frozen)
- Legumes/pulses (e.g., beans, lentils, chickpeas)
- Nuts or nut spread (unsalted)
- Unprocessed fish
- Unprocessed poultry
- Unprocessed red meat
- Unprocessed vegetarian alternatives (e.g., tofu, tempeh, seitan)
- Whole wheat
- Milk
- Other dairy products (e.g., yoghurt, cheese)
- Plant-based drinks (e.g., almond, oat, soy, rice)
- Non-sugared beverages (e.g., water, coffee, tea)

*Nutrient-Poor Food Intake*
- Processed meat
- Sweet snacks (e.g., sweets, cookies, cakes, pies)
- Salty snacks (e.g., crisps, salted nuts)
- White wheat
- Sugared beverages (e.g., soft drinks, sugared coffee/tea)
- Alcoholic beverages

### Food Advice before or during the COVID-19 Lockdown
Question: Whose food advice do you listen to?
(1 = Never; 7 = Every time I need or want advice)
- Family members
- Friends
- Experts (e.g., dietitians, nutritionists, medical doctors, scientists)
- Celebrities (e.g., celebrity chefs, celebrities, and food influencers).

### Sociodemographic Characteristics
*Gender*
- Female (0)
- Male (1)

*Age*
(Ranging from 18 to 120)

*Geographic location*
- Mainland China (1)
- Non-Mainland China (Taiwan and Macao) (0)

*Education*
- Below high school diploma (1)
- High school diploma or equivalent (2)
- Bachelor's degree (3)
- Master's degree (4)
- Doctorate (5)

*Employment status*
- No work (0)
- Work (1)

**COVID-19-Related Environmental Factors**
*Income loss*
Question: Have you lost (a part of your) income since the lockdown?
- Yes (0)
- No (1)

*Financial difficulties for food purchase*
Question: In general, how often is it a struggle to have enough money to go shopping for food?
- Never (1)
- Very rarely (2)
- Rarely (3)
- Sometimes (4)
- Frequently (5)
- Very frequently (6)
- Every time I go shopping for food (7)

*Degree of closure measures*
Question: Which of the following lockdown measures are currently in place?
(Multiple choice: 0 = No; 1 = Yes)
- Events are suspended
- Restaurants are closed for dining in
- Bars and pubs are closed
- Most non-essential shops are closed
- Schools are closed
- Public gatherings are prohibited (not allowed)
- Public gatherings are restricted (allowed under restrictions)
- If possible, people need to work from home
- People in elderly homes are not allowed visitors/ only a restricted number of visitors
- Non-essential movement is banned
- Private gatherings are prohibited (people cannot visit other people)
- Private gatherings are restricted (people can visit other people, but under restrictions)
- Country borders are closed
- Non-essential production has stopped
- Face masks are mandatory in public

***Self-reported lockdown time***
Question: How many weeks have you been in lockdown?
(Ranging from 1 to 50)
